# *In vitro* bond strengths post thermal and fatigue load cycling of sapphire brackets bonded with self-etch primer and evaluation of enamel damage

**DOI:** 10.4317/jced.56444

**Published:** 2020-01-01

**Authors:** Ali I. Ibrahim, Noor R. Al-Hasani, Van P. Thompson, Sanjukta Deb

**Affiliations:** 1 Centre for Oral, Clinical and Translational Sciences, Faculty of Dentistry, Oral & Craniofacial Sciences, King’s College London, London, UK; 2 Department of Orthodontics, College of Dentistry, University of Baghdad, Baghdad, Iraq; 3 Institute of Pharmaceutical Science, King’s College London, London, UK; 4 Department of Basic Sciences, College of Dentistry, University of Baghdad, Baghdad, Iraq

## Abstract

**Background:**

This *in vitro* study compares a self-etch primer (SEP) to an etch-and-rinse (EaR) for bonding sapphire brackets by evaluation of the enamel etch-pattern, shear bond strength, amount of remnant adhesive and enamel surface damage following thermal and fatigue cyclic loading.

**Material and Methods:**

Ceramic (sapphire) brackets were bonded to 80 extracted human premolars using two enamel etching protocols: conventional EaR using 37% phosphoric acid (PA) gel (control), and a SEP (Transbond Plus). Each group was subdivided into two subgroups (n=20 teeth) according to the time of bracket debonding: after 24 h water storage or following 5000 thermo-cycles plus 5000 cycles fatigue loading, to determine the shear bond strength (SBS), adhesive remnant index (ARI score), with scanning electron microscopy (SEM) evaluation of enamel condition.

**Results:**

The control subgroups consistently exhibited significantly higher (*p*<0.05) SBS mean values (23.4-29.8 MPa) than the SEP subgroups (15.1-22.4 MPa) at both bracket debonding time points. However, the SEP subgroups yielded milder etch-patterns and attained SBS values above the minimum requirement range for clinical performance. In addition, the higher SBS of control subgroups was accompanied with higher ARI scores and enamel damage grades than SEP subgroups as confirmed by SEM. Thermocycling and fatigue significantly reduced the SBS of all subgroups, with a non-significant drop in the amount of adhesive residue or enamel damage.

**Conclusions:**

The use of SEP can be a suitable alternative to the conventional PA gel for sapphire bracket bonding as it maintains suitable bond strength and has the potential to produce both less remnant adhesive and enamel damage.

** Key words:**Enamel etching, ceramic brackets, orthodontic bonding, adhesive remnants, enamel damage.

## Introduction

Correction of mal-aligned teeth and/or jaw discrepancies using fixed orthodontic appliances can improve the facial aesthetics and oral function, resulting in a profound effect on both the psychological well-being of the person and dental health. Attachment of a fixed orthodontic appliance to the enamel surface of teeth entails enamel conditioning with an acid followed by bonding of metal or ceramic brackets. Research has shown that an etch-and-rinse (EaR) approach using phosphoric acid remains the preferred choice for enamel conditioning in orthodontics since it guarantees a durable bracket bond to enamel (1,2). However, high shear bond strength (SBS) values have been accompanied by more adhesive residues with iatrogenic enamel fracture, chipping, or cracking due to the higher debonding force required to remove the brackets after treatment (3). Attempts to employ various concentrations of other acids such as pyruvic, lactic, nitric, maleic, tannic and citric acids resulted in suboptimal bond strengths not suiTable for clinical performance and failed to outperform the etching ability of phosphoric acid (4). On the other hand, recent attempts to shorten the chair-side treatment time and minimize enamel damage involved the use of self-etch primers (SEP), which combine enamel etching and priming in one step, as an alternative to the standard EaR approach (5). SEPs have the advantages of time saving, simultaneously demineralizing and infiltrating the tooth surface to the same depth, theoretically ensuring complete penetration of the adhesive into the etched enamel surface. However, there is controversy in the literature regarding the SBS, amount of remnant adhesive and degree of enamel damage following the use of SEP due to the lack of standardized testing methods (6,7). Significant enamel cracking at bracket debonding has been reported by studies that claimed suiTable SBS after enamel conditioning with strong SEP (7,8); whilst milder SEPs were less effective in enamel etching and resulted in a poor hybridization phase (which involves infiltration and subsequent in situ polymerization of resin within the created surface micro-porosities) (5,9).

The use of ceramic brackets in orthodontics is increasing due to their superior aesthetics compared to metallic brackets and patient requests for less visible orthodontics. Ceramic brackets are made of polycrystalline or monocrystalline (difference is in their optical clarity) forms of aluminum oxide, which is an inert material (10). Sapphire brackets, which bond to the enamel surface depending on mechanical retention, represent the newest and most aesthetically accepTable form of ceramic brackets. Synthetic sapphire is a single crystal form of corundum, aluminum oxide (Al2O3), which is in the purest form with no porosity or grain boundaries (11). High enamel bond strengths coupled with high stiffness of sapphire brackets and lack of ductility, however, are the main problems associated with bracket removal. Particularly, the resistance to deformation can cause stress build-up at the enamel-adhesive-bracket interfaces during bracket removal, increasing the risk of ceramic bracket fracture, enamel cracks and tear-outs (10,12,13).

Previous *in vitro* studies related to use of SEP have focused on investigating the performance of metallic brackets and frequently assessed the SBS and remnant adhesives over a short time period, i.e. within 30 minutes and/or few days of storage in distilled water after the bracket bonding procedure (8,13,14). There is consensus on the importance of adopting rigorous testing methods (e.g. fatigue and thermocycling) that can simulate the complex intraoral conditions, hence mimicking the long-term clinical survival of brackets. The testing methods premised on a short-term assessment do not factor the effects of the daily exposure of the tooth-adhesive-bracket interfaces to the mechanical and thermal changes occurring in the oral cavity throughout orthodontic treatment (15). The effects of exposing ceramic brackets bonded with SEP to both thermocycling and fatigue (cyclic loading) tests before bracket debonding on bond strength, remnant adhesive and enamel surface integrity have not been reported previously. Therefore, this study aims to determine the SBS, amount of remnant adhesive, enamel etch-patterns, and evaluate the enamel damage of highly aesthetic ceramic brackets (monocrystalline sapphire) bonded with a SEP following exposure to rigorous thermocycling and fatigue tests in comparison with the standard EaR technique. The tested hypothesis was whether sapphire bracket bonding with a SEP could eliminate the remnant adhesive and enamel damage after debonding, without compromising the bond strength.

## Material and Methods

Eighty extracted human premolar teeth were collected from adolescent and young patients (12-25 years) to conduct *in vitro* bonding procedures, after acquiring ethical approval from the National Research Ethics Service Committee London-Riverside (REC Reference 14/LO/0123). After extraction, the teeth were cleaned in running water, then stored in a 1% chloramine-T trihydrate bacteriostatic/bactericidal solution for a maximum of one week and thereafter stored in distilled water (ISO/TS 11405:2015). Criteria for tooth selection included: intact buccal enamel surface without cracks and caries (examined under stereomicroscope x10 magnification), with no history of previous orthodontic or bleaching treatments (12). Teeth were randomly divided into two equal groups according to the enamel conditioning protocol: EaR (control) and SEP (experimental).

-Sample preparation for bracket bonding 

Teeth were mounted in acrylic blocks using rubber moulds (14 mm length x14 mm width x17 mm depth). Teeth were aligned so that the middle third of the buccal surface was parallel to the analyzing rod of a surveyor, to ensure a debonding force running parallel to the bonded bracket base. Then self-cure clear acrylic (Oracryl, Bracon, UK) was poured around the tooth up to about 1 mm apical to the level of cemento-enamel junction. The mounted teeth were subsequently stored in distilled water at lab temperature until bonding.

-Enamel conditioning and bracket bonding procedures 

The conventional EaR protocol (1) was followed using 37% phosphoric acid (PA) gel (Orthotechnology, USA) as the control etchant. Starting with polishing (10 s) using pumice slurry and rotary rubber cups, water irrigation (10 s) and oil-free air dryness (10 s), the 37% PA etchant gel was applied on the middle third of the buccal surface for 30 s, irrigated with water for 20 s and dryness for 20 s. A thin layer of Transbond XT light-cure primer (3M Unitek, Monrovia, California, USA) was applied onto the etched surface and spread by air-jet (3 s) so that the tooth was ready for bracket bonding.

For the experimental group, a self-etch primer (Transbond Plus, supplied by 3M Unitek as lollypops, Monrovia, USA) was used according to the manufacturer instructions. After pumicing and dryness of the enamel surface (as described for the control group), the SEP was gently rubbed on the buccal enamel surface for approximately 3 seconds using the disposable applicator supplied with the lollypop, followed by a gentle burst of moisture-free air to the enamel, ready for bracket bonding.

Following the two aforementioned etching protocols, each bracket base was loaded with the adhesive material and attached to the enamel surface. Bonding of all brackets was conducted using Transbond XT light-cure composite adhesive (3M Unitek, Monrovia, California, USA). Pre-adjusted upper premolar ceramic brackets (NeoCrystal, monocrystalline sapphire, MBT, slot 0.022x0.028 inch, Henry Schein, USA) were used for bonding all teeth. LED Light curing (3M ESPE, Elipar DeepCure-S, USA, 1470 mW/cm2 light intensity) was applied for 20 s (10 s on each mesial and distal side) according to the manufacturer instructions. After bracket bonding, the bonded teeth were stored in distilled water at 37oC for 24 h. Half of the samples (n=20 per subgroup) were debonded at 24 hours and the bond strengths and ARI scores between the control and SEP groups were compared. The other half of the specimens were subjected to thermocycling and then these were further subjected to fatigue (as will be described later).

-Bracket debonding for SBS and adhesive remnant index (ARI) assessment

The 24 hours SBS testing was conducted using a chisel on a universal testing machine (Instron, Model 5569A, USA) with an occluso-gingival load applied vertically at the bracket base at a crosshead speed of 0.5 mm/min. The SBS values were calculated in MPa by dividing the load at failure by the bracket base surface area. The debonded teeth were examined with a stereomicroscope (MEIJI, EMZ-TR, Japan) under x10 magnification for the amount of remnant adhesive left, and scored according to the ARI scoring system (16): Score 0: No adhesive left on the tooth, Score 1: Less than half of the adhesive left on the tooth, Score 2: More than half of the adhesive left on the tooth, Score 3: All adhesive left on the tooth, with a distinct impression of the bracket mesh. This same approach was utilized on the debonded specimens after the fatigue testing as was the SEM analysis of the enamel.

-Confocal laser scanning microscopy (CLSM) samples

CLSM was carried out to compare the etch-pattern produced by the self-etch primer to 37% PA gel. Five extracted, non-cracked and caries-free human molars were used for obtaining flat, wide buccal enamel surfaces following a previously described protocol (17). Before enamel etching, 0.1 wt.% Rhodamine B dye (Sigma–Aldrich, UK) was added to the control 37% PA gel and the self-etch primer to obtain fluorescent etchants. Three flat-surface enamel samples were used for CLSM examination; the surface of each sample was divided using adhesive tapes into 3 zones: one un-etched enamel, and two zones to be etched with 37% PA gel or SEP according to the protocols described above. Etched samples were kept dry at ambient laboratory conditions for 24 h before examination. The microscopy examination was performed using a CLSM (Leica SP2 CLSM; Leica, Heidelberg, Germany) equipped with a 63x magnification/1.4 NA (numerical aperture) oil-immersion lens, and a laser illumination setting of 568-nm krypton (rhodamine excitation). Representative images of the most common distinguishing characteristics detected in each specimen were captured. All images were further reconstructed with Image J software.

-SEM analysis of enamel post debonding of brackets

Three random samples of each subgroup were further sputter-coated with gold palladium (15 nm) and examined using SEM machine (Hitachi High Technologies, S-3500N) at an accelerating voltage of 10 kV. The crown of each tooth was sectioned mesio-distally through the occlusal central fossae using a diamond wafering blade to obtain the buccal bracket-debonded half, sputter-coated with gold palladium and examined with SEM. The debonded enamel surface was assessed according to the enamel damage index (EDI) which includes the following categories (18): Grade 0: Smooth surface without scratches, and perikymata might be visible; Grade 1: AccepTable surface, with fine scattered scratches; Grade 2: Rough surface, with numerous coarse scratches or slight grooves visible; Grade 3: Surface with coarse scratches, wide grooves, and enamel damage visible to the naked eye.

-Thermocycling Procedure: bracketed premolars subjected to 5000 thermo-cycles 

Samples were put inside a perforated stainless steel container and thermo-cycled between hot (55°C) and cold (5°C) water baths for 4 continuous days (5000 cycles) with a dwell time of 30 s in each bath and transfer time of 5 s (19). A robot arm was used to transfer the samples between the two baths. Once completed, this thermocycling was followed by the fatigue load cycling procedure.

-Fatigue (F) Procedure: bracketed premolars subjected to 5000 cyclic loadings 

To run the fatigue test, the acrylic block of each specimen was fixed within the grips of a fatigue machine (Bose, EnduraTec-3300, Eden Prairie, MN, USA) using a metal clamp. A hardened stainless steel chisel (0.5 mm) was adjusted to apply an occluso-gingival force to the base of the bracket.

Fatigue testing (20) was started with an initial load value that represents 60% of the mean static SBS of the corresponding subgroup obtained by bracket debonding carried out after 24 h water storage of the bonded teeth. The cyclic testing was achieved at a frequency of 1 Hz, which corresponds to the reported oral chewing frequency (21). The cyclic loadings were conducted sequentially for each subgroup, with the maximum applied stress in each succeeding sample being increased or decreased by 5% of the initial load applied, according to whether the previous stress resulted in a failure or non-failure of the bracket.

-Statistical Methods

Analysis was conducted using SPSS statistical software (version 22, SPSS Inc., IBM, Chicago, USA). Data were tested for normality using Histogram/Q-Q plots/Shapiro-Wilk tests. Independent samples t-test was conducted for parametric data analysis (SBS), while Mann-Whitney test was carried out for non-parametric data analysis (ARI score). All statistical analyses were conducted at a level of significance *P*> 0.05.

## Results

-Shear bond strength (SBS) and adhesive remnant index (ARI)

For both bracket debonding time points (24 h WS and following TC and F), the control subgroups consistently exhibited significantly higher SBS mean values in comparison with SEP subgroups. However, on examination of the enamel surfaces post debonding of the brackets, the SEP subgroups exhibited less amounts of remnant adhesive left on enamel (83% ARI scores of 1 and 2) as compared with the control subgroups (76% ARI scores of 2 and 3), but the difference was not statistically significant ( Table 1).

**Table 1:**
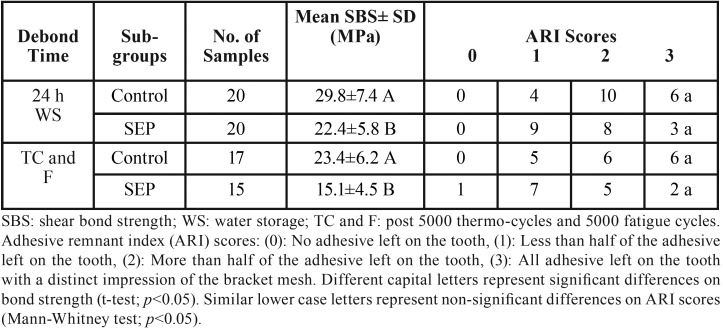
Shear bond strength and adhesive remnant index scores of ceramic bracket debonding of two etching protocols after 24 hour water storage, and post 5000 thermo-cycles and fatigue (maximum 5000 cycles).

In comparison with bracket debonding following 24 h WS, the aggressive thermocycling and fatigue load cycles resulted in many bracket failures before starting the bracket debonding procedure. The control subgroups encountered three bracket failures (one bracket during TC and two brackets failed in cyclic loadings), while five brackets failed from the SEP subgroups (two in TC and three during cyclic loading). The thermocycling and fatigue significantly reduced the SBS of the control and SEP subgroups, with a non-significant drop in the amount of adhesive residue (ARI scores) as shown in  Table 2.

**Table 2:**
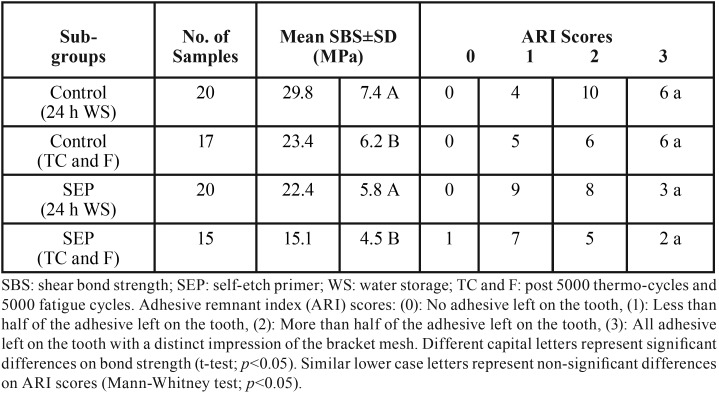
Comparison of shear bond strength and adhesive remnant index obtained at bracket debonding of sapphire brackets after 24 hour water storage and post thermocycling and fatigue.

CLSM analysis of enamel etch-patterns (flat molar buccal surface, no bracket bonding)

**Figure 1:**
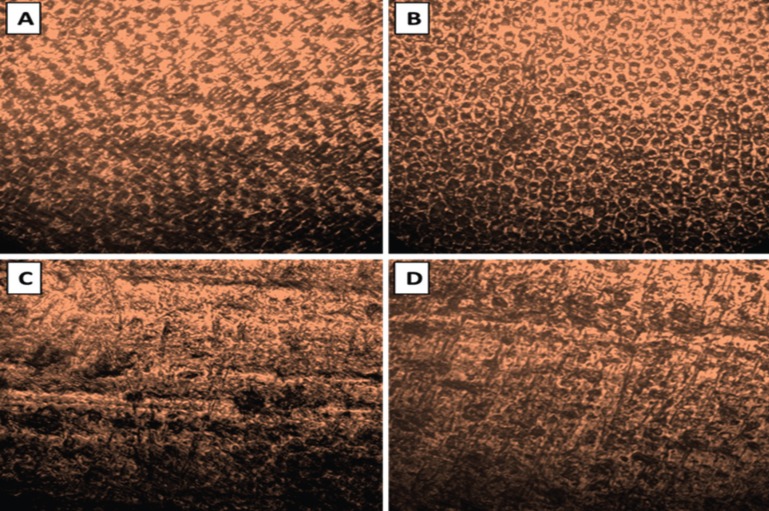
Confocal laser scanning images (63x magnification) of flat, highly polished buccal enamel surfaces. A and B: Enamel etching with 37% PA gel produced typical honeycomb etch-patterns with a uniform distribution of the micro-pores; C and D: Etching with the self-etch primer yielded a milder, ill-defined etch-pattern with diffuse distribution of micro-pores.

Enamel etching with 37% PA gel yielded the typical etch-pattern (honeycomb appearance) caused by the preferential dissolution of the enamel prism cores, whilst etching with SEP resulted in a less discernible etch pattern showing areas of unaffected enamel with production of diffuse honeycombs and disperse micro-pores as shown in Figure 1.

SEM analysis of debonded enamel surfaces at 24 h water storage and following TC and F 

**Figure 2:**
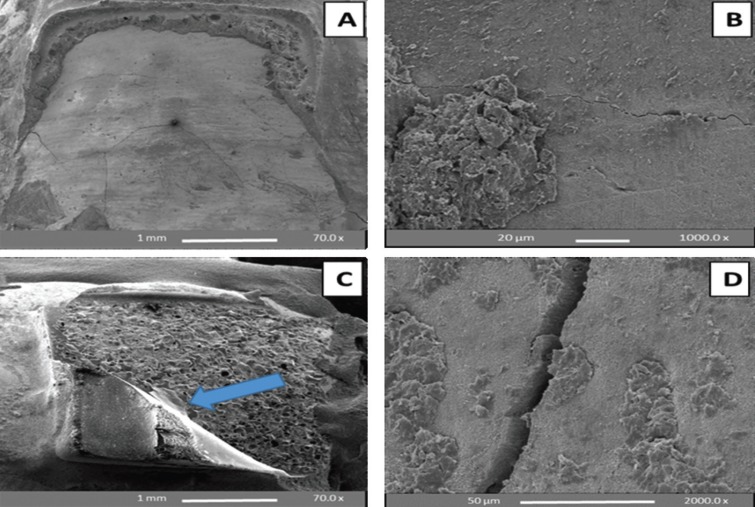
SEM images of premolar enamel surfaces etched with SEP and de-bracketed after 24 h water storage (A, B) and following thermocycling and fatigue (C, D). A and C: Top view of the whole buccal surface showing minimal adhesive left with enamel cracks (A) and complete retention of adhesive with bracket fracture (arrow in C); B and D: At high magnification, etching with SEP yielded regular, un-roughened surface, but could not eliminate remnant adhesive and cracks.

**Figure 3:**
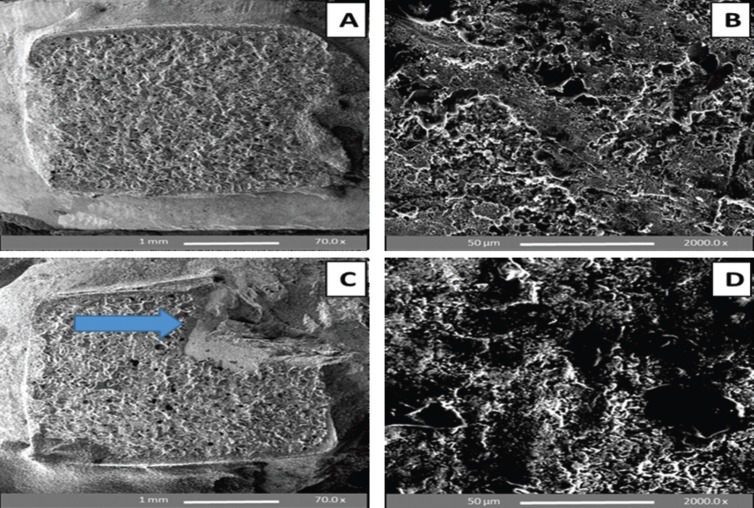
SEM images of premolar enamel surfaces etched with 37% phosphoric acid (PA) gel and de-bracketed after 24 h water storage (A, B) and following thermocycling and fatigue cycles(C, D). A and C: Top view of the entire buccal surface showing complete retention of adhesive in addition to enamel chipping (arrow in C); B and D: At high magnification, etching with PA gel resulted in rough, irregular and grooved enamel surface after sapphire bracket debonding.

The SEM results confirmed the retention of adhesive (mainly scores 2 and 3 exhibited by the control, whilst scores of 1 and 2 were obtained using the SEP) and enamel damage (cracking or chipping) was observed in all subgroups both at 24 h WS bracket debonding and following TC and F procedures (Figs. 2,3). According to the enamel damage index (EDI), enamel etching with 37% PA gel (control) yielded a mix of grades 2 and 3 (generally rough surface with numerous coarse scratches, grooving or cracking) following debonding of the sapphire brackets. On the other hand, enamel etching with SEP yielded a mix of grades 1 and 2 (generally less roughness with fine scattered scratches, grooving or cracking), i.e. less remnant adhesive and enamel damage.

## Discussion

Despite the significantly successful outcome of orthodontic therapy, many patients persist to refrain from this treatment for various reasons. While the long treatment duration and difficulties in maintenance of proper oral hygiene are common obstacles to various age group patients, the unaccepTable aesthetics of metallic orthodontic brackets presents a special concern for adult patients. The invention of ceramic brackets enables to overcome this issue of aesthetics that has had an impact on orthodontic treatment cases. However, with the advent of aesthetic ceramic brackets, the concern has been the damage of the enamel during bracket removal due to the high bond strengths between the bracket and enamel (11,22). Thus, lowering the bond strength, whilst keeping it within a clinically accepTable range, and leaving no or minimal adhesive remnants to eventually minimize enamel damage constitute the most desirable properties for a successful bonding system, yet very challenging to attain.

A minimum bond strength range of 6-10 MPa has been suggested to be suiTable for bracket bonding towards an acceptable clinical performance (7). The common practice before bracket bonding involves etching the enamel with 35-40% phosphoric acid (the conventional EaR approach) to allow preferential dissolution of the superficial enamel (decalcification) that creates micro-porosities on the surface to enable ingress of resin monomers that upon polymerization become micro-mechanically interlocked. The bond strengths obtained via this protocol of bracket bonding are usually high, ranging between 9-35 MPa, with usually higher values attained for ceramic than metal brackets (6,23). The SBS results obtained for all subgroups in this study were within the confines of this range. However, enamel etching with 37% PA gel consistently resulted in significantly higher SBS values than etching with the SEP, but with more enamel damage and remnant adhesive left on enamel after bracket removal. While this finding is in agreement with the fact that the 37% PA etchant yields the highest bond strengths in comparison to any other etchant, this unnecessarily excessive bond strength has been a point of contention as it is frequently accompanied by varying forms of enamel damage (8,24). In addition, since the debonding inevitably leaves adhesive remnants on the enamel surface, enamel clean-up and polishing is required to remove these remnants (usually using rotary burs) resulting in increased chair-side time and enamel scratching (3,7).

On the other hand, although enamel etching with SEP exhibited lower SBS (15.1-22.4 MPa) than the PA gel (23.4-29.8 MPa) at both bracket debonding time points, the values obtained were well above the minimum requirement for clinical performance (6-10 MPa). Moreover, this was accompanied with less ARI scores and EDI grades that are certainly advantageous. It has been reported that SEPs can produce shorter and thinner resin tags than the EaR approach, hence exhibit lower SBS and may leave less remnant adhesive (24,25); yet insufficient information is available about the enamel etch-patterns they produce. In this study, the etch-pattern attained following treatment with a SEP was examined using the confocal laser scanning microscopy with a fluorescent dye technique for the first time. The CLSM can provide evidence about the enamel etching effects induced by acids depending on the degree of image fluorescence, which increases with the greater loss of organic matrix and exposure of enamel prisms (26). This is achieved through the use of fluorescent dyes which promote certain emission wavelengths when they are excited by laser with specific wavelengths, hence can be traced at their locations in a material and/or tissue at dilute concentrations (17). The CLSM images showed that enamel treatment with the PA gel or SEP resulted in a visible increase in fluorescence, evidenced by the more apparent honeycomb morphology, which was more discernible with an apparently greater loss of organic matrix in areas where the etched enamel prisms were clearly exposed. Enamel etching with 37% PA gel produced the typical honeycomb etch-pattern with a uniform distribution of the micro-pores, whilst etching with the SEP yielded an ill-defined pattern with disperse islands of micro-pores and areas of unaffected enamel indicating a milder etching effect, which can be attributed to the higher pH value of the SEP than the 37% PA gel (5).

For each control and SEP subgroup, the thermocycling and fatigue procedures resulted in a significant drop in SBS as compared with the 24 h WS bracket debonding; however, the outcomes were almost consistent at both bracket debonding time points in terms of the amount of remnant adhesive and enamel damage produced. Thermal cycling and cyclic fatigue have been recognised as aggressive and extremely crucial artificial tests in predicting the long-term intraoral survival of an orthodontic bonding system (15). Thermocycling has been shown to decrease bond strength between 20-70%. This artificial aging test induces mechanical stresses initiated by differential thermal changes that can directly stimulate crack propagation through bonded interfaces (27). Further to the weakening effect induced by thermocycling, this was followed by fatigue load cycling, which represents the failure or degradation of mechanical properties after repeated loading at a level well below the ultimate fracture strength of the material or interface (20). The use of a high initial cyclic loading (60% of the static SBS) and high cycle number (5000 cycles) resulted in 5 bracket failures (out of 40 bonded teeth) in this study. A similar fatigue protocol utilized in previous *in vitro* studies resulted in a significant drop in SBS of metallic brackets following the use of EaR protocol (21) and SEP protocol (28). Therefore, the successive exposure to both thermo-cycling and fatigue stands for the significant drop in SBS of both control and SEP subgroups in this study. However, non-significant differences in the distribution of ARI scores and EDI grades were exhibited by all subgroups when comparing the results of TC and F experiments to those of 24 h water storage. Different studies have shown that the mode of bracket failure is not affected significantly by ageing tests whether using the EaR or self-etch approach, given that both the enamel-adhesive and bracket-adhesive interfaces have almost similar strengths (19,25).

It has been reported that the risk of enamel damage upon debonding increases as the bracket bond strength exceeds 14 MPa since excessive bond strengths entail extra force application to debond a bracket, increasing the chance of damage to the enamel (7,8). This agrees with a recent recommendation to keep the maximum bond strength below the mean tensile strength of enamel (17), reported to be nearly 14.5 MPa with a range of 10-25 MPa according to the orientation of enamel prisms (29). While the conventional EaR method is based on micro-mechanical retention between the adhesive and enamel, the SEP approach involves an additional chemical interaction between functional monomers and tooth substrate components. The 10-methacryloyloxydecyl dihydrogen phosphate (10-MDP) molecule, which is the strong functional monomer in Transbond Plus self-etch primer, chemically bonds to the calcium of enamel hydroxyapatite forming sTable calcium-phosphate salts, along with only a limited surface-decalcification effect (30). It is this additional chemical bonding that may account for the inability of SEP subgroups to significantly reduce the remnant adhesive and enamel damage encountered at sapphire bracket debonding in this study.

## Conclusions

The use of a self-etch primer for simultaneously etching and priming the enamel reduced, but could not completely eliminate, the enamel damage and amount of remnant adhesive left on enamel after sapphire bracket debonding. Exposure of sapphire brackets bonded with a SEP to thermocycling and then cyclic loading reduced the bond strength, yet it was within the range suiTable for clinical performance. The shorter bonding time combined with the potential to produce less remnant adhesive and enamel damage render the SEP approach a suiTable alternative to the aggressive PA gel for enamel conditioning before sapphire bracket bonding.
